# NDUFA4L2 in smooth muscle promotes vascular remodeling in hypoxic pulmonary arterial hypertension

**DOI:** 10.1111/jcmm.16193

**Published:** 2020-12-19

**Authors:** Yun Liu, Xiaowei Nie, Jinquan Zhu, Tianyan Wang, Yanli Li, Qian Wang, Zengxian Sun

**Affiliations:** ^1^ Department of Pharmacy The First People's Hospital of Lianyungang Lianyungang China; ^2^ Department of Pharmacy The Affiliated Lianyungang Hospital of Xuzhou Medical University/The First People's Hospital of Lianyungang Lianyungang China; ^3^ Institute for Hepatology National Clinical Research Center for Infectious Disease Shenzhen Third People's Hospital Shenzhen China; ^4^ Lung Transplant Group Wuxi People's Hospital Affiliated to Nanjing Medical University Wuxi China; ^5^ Department of Anesthesiology Children's Hospital of Soochow University Suzhou China

**Keywords:** hypoxia, NDUFA4L2, proliferation, pulmonary arterial hypertension, pulmonary vascular remodelling

## Abstract

Pulmonary arterial hypertension (PAH) is characterized by a progressive increase in pulmonary vascular resistance and obliterative pulmonary vascular remodelling (PVR). The imbalance between the proliferation and apoptosis of pulmonary artery smooth muscle cells (PASMCs) is an important cause of PVR leading to PAH. Mitochondria play a key role in the production of hypoxia‐induced pulmonary hypertension (HPH). However, there are still many issues worth studying in depth. In this study, we demonstrated that NADH dehydrogenase (ubiquinone) 1 alpha subcomplex 4 like 2 (NDUFA4L2) was a proliferation factor and increased in vivo and in vitro through various molecular biology experiments. HIF‐1α was an upstream target of NDUFA4L2. The plasma levels of 4‐hydroxynonene (4‐HNE) were increased both in PAH patients and hypoxic PAH model rats. Knockdown of NDUFA4L2 decreased the levels of malondialdehyde (MDA) and 4‐HNE in human PASMCs in hypoxia. Elevated MDA and 4‐HNE levels might be associated with excessive ROS generation and increased expression of 5‐lipoxygenase (5‐LO) in hypoxia, but this effect was blocked by siNDUFA4L2. Further research found that p38‐5‐LO was a downstream signalling pathway of PASMCs proliferation induced by NDUFA4L2. Up‐regulated NDUFA4L2 plays a critical role in the development of HPH, which mediates ROS production and proliferation of PASMCs, suggesting NDUFA4L2 as a potential new therapeutic target for PAH.

## 1 INTRODUCTION

Pulmonary artery hypertension (PAH) is a type of cardiovascular disease with a high degree of malignancy. The diagnostic criteria is based on the 2018 6th World Pulmonary Hypertension Conference (WSPH) redefining the definition of pulmonary hypertension (PH) as mPAP >20 mmHg measured by the right heart catheter at rest. In addition, the definition of all precapillary PH also includes pulmonary vascular resistance ≥3 Wood units.^1^ The worsening symptoms and poor prognosis caused by the development of PAH are associated with elevated pulmonary arterial pressure and right ventricular hypertrophy (RVH) eventually leading to right heart failure.^2^ Long‐term oxygen therapy can effectively prolong survival, but can only slightly reduce pulmonary arterial pressure. So far, no targeted drugs including pulmonary vasodilators have shown effective therapeutic effects in the treatment of PAH.^3^ Although the natural survival time of patients after diagnosis has been increased from the original 2.8‐6 years, it still causes a huge clinical and economic burden. Although the number of PAH‐related hospital admissions decreased between 2001 and 2012, the average cost and length of hospital admissions per PAH‐related admissions increased, and hospital mortality did not decrease significantly.^3^ Therefore, it is important to understand the pathogenesis of PAH and find early diagnosis, prognostic judgment and new molecular markers.

The aetiology of PAH involves environmental and genetic factors, and its pathogenesis is complex, so far there is no complete explanation. Hypoxia is an important cause. Long‐term exposure to hypoxic conditions can cause inflammation, vasoconstriction, smooth muscle cell proliferation, muscleization of the anterior capillaries and loss of distal pulmonary vessels, which are key pathophysiological processes of PAH.

After the pulmonary artery is subjected to various injuries or hypoxia, the vascular wall tissue structure and its function undergo pathological changes, mainly including the two pathological processes of pulmonary angiogenesis and pulmonary artery middle smooth muscle thickening. Although thickening of all layers of the pulmonary vessel wall intima, media and adventitia can cause PAH, vascular remodelling caused by medial thickening plays a major role in its development. In the pulmonary blood vessels, pulmonary artery smooth muscle cells (PASMCs) are the main cells constituting the pulmonary artery wall. Their hypertrophy is an important pathological feature of hypoxic pulmonary vascular structural changes. When PAH occurs, PASMCs proliferation increases, apoptosis decreases, and DNA synthesis increases. These abnormal changes promote thickening of the pulmonary artery wall and narrowing of the lumen, which in turn leads to increased pulmonary vascular resistance, RVH and continuous increase in pulmonary artery pressure.^4^ Therefore, further searching for new targets for anti‐PASMCs proliferation and hypertrophy, and exploring related intracellular molecular mechanisms have important scientific value and clinical application prospects.

Numerous studies have showed that oxidative stress plays a crucial role in the development of PAH.^5^, ^6^ Whether in the animal model of PAH induced by SU5416‐hypoxia^7^ or in the rat PAH model induced by hypoxia^8^ and PAH patient specimens,^9^ reactive oxygen species (ROS) and other oxidative stress indicators have increased significantly. In particular, the release of ROS from mitochondria caused by hypoxia attacks and promotes the oxidation of polyunsaturated fatty acids, a process called lipid peroxidation.^10^, ^11^ Lipid peroxidation produces a variety of oxidation products, with aldehydes being the major end products. Toxic aldehydes are highly reactive and interact with cellular macromolecules (including nucleic acids and proteins) to produce a variety of adducts, resulting in DNA damage and protein inactivation. Among the products of lipid peroxidation, 4‐hydroxynonene (4‐HNE) is considered the most toxic aldehyde and malondialdehyde (MDA) appears to be the most mutagenic aldehyde.^12^ Lipid oxidation has been reported to participate in the proliferation of PASMCs and exacerbate the development of PAH.^12^ However, it remains uncertain whether the production of toxic aldehydes contributes to hypoxic pulmonary vascular remodelling (PVR).

NADH dehydrogenase (ubiquinone) 1 alpha subcomplex 4 like 2 (NDUFA4L2), as part of the electron transport chain (ETC) complex I (complex I) subunit, belongs to the NDUFA4 subunit family of the complex I subunit, can fine‐tune the activity of complex I, thereby mediating mitochondria to activate oxidative phosphorylation and produce ROS.^13^ NDUFA4L2, which is one of the important components of the ETC complex I subunit in mitochondria, is an important site for ROS generation and is widely involved in the regulation of biological processes. For example, NDUFA4L2 is abnormally expressed in many types of cancer, including malignant hepatocellular carcinoma,^14^ clear cell renal cell carcinoma^15^, ^16^ and colorectal cancer.^17^ NDUFA4L2 inactivation increases mitochondrial activity and oxygen consumption, leading to accumulation of ROS and apoptosis in hepatocellular carcinoma.^14^ NDUFA4L2 has been reported to promote hypoxic cell proliferation by increasing mitochondrial ROS and nucleic acid production.^18^ However, the molecular mechanism of hypoxic‐induced PASMCs proliferation is unknown, and the role of NDUFA4L2 in vascular remodelling of PAH has not been reported.

In the present study, we found that the production of 4‐HNE was increased in the plasma of PAH patients and chronic hypoxia‐induced PAH rat animal models. Using multiple approaches, we have demonstrated that inhibition of NDUFA4L2 reduced 4‐HNE levels and attenuated the severity of hypoxic PAH. NDUFA4L promoted the proliferation of PASMCs by regulating the upstream HIF1α pathway and the downstream p38‐5‐lipoxygenase (5‐LO) signal, thereby promoting PVR and inducing PAH.

## 2 MATERIAL AND METHODS

### 2.1 Animal models

Adult Wistar male rats with an average weight of 200 g were obtained from the Experimental Animal Center of Xuzhou Medical University (Grade II). Rats were either placed in normal air or in a normobaric hypoxic chamber (FiO_2_ 10%) for 3 weeks. All animal procedures were in accordance with the National Institutes of Health Guidelines and were approved by the Animal Use and Care Committee of the First People's Hospital of Lianyungang. To understand the role of NDUFA4L2 in hypoxia‐induced PAH. Adult Wistar male rats were randomly divided into four groups (n = 6). Chemically modified anti‐small interfering RNA (siRNA) oligonucleotides targeting NDUFA4L2 (siNDUFA4L2) or nontargeting control (siNC) were administered intraperitoneally for two times a week for 3 weeks in the chronic hypoxia‐induced PAH rats. The primer sequences were designed and synthesized by Ribobio co., LTD based on the mRNA sequences obtained from the NCBI database as follows: si‐r‐NDUFA4L2:GTTTCCACCGACTACAAGA. Wistar male rats treated with siNC were fed in normal air or in a normobaric hypoxic chamber (FiO_2_ 10%) for 3 weeks. Wistar male rats treated with siNDUFA4L2 were fed in normal air or normal pressure hypoxic chamber (FiO_2_ 10%) for 3 weeks.

### 2.2 Clinical samples collection

Human lung samples of PAH were obtained from the Lung Transplant Group, Affiliated Wuxi People's Hospital of Nanjing Medical University (Wuxi, China). Paired healthy controls were collected from the donors not suitable for transplantation. Each individual gave written informed consent prior to their participation. This study was approved by Wuxi People's Hospital of Nanjing Medical University and Xuzhou Medical University Affiliated Lianyungang Hospital which was in accordance with the Code of Ethics of the Helsinki Declaration of World Medical Association for use of Human Samples (YJ‐20200208001).

### 2.3 Haemodynamic experiments

Right ventricular systolic pressure (RVSP) was analysed as previously described.^19^ Rats were anaesthetized with 3% pentobarbital sodium 30 mg/kg by intraperitoneal injection. After anaesthesia, the right jugular vein was peeled off, and a heparin anticoagulated PV‐I polyethylene catheter (curved tip) was inserted into the right ventricle (RV) through the right jugular vein, followed by blood into the pulmonary arteries (PAs). Pulmonary arterial pressure was analysed by a 1.4 F pressure transducer catheter (Millar Instruments) and AcqKnowledge software (Biopac Systems Inc).^20^ After analysing the hemodynamic data, the rat thorax was opened. The lungs and heart were moved together into a petri dish filled with cold PBS. The ratio of RV/left ventricle + septum (RV/LV + S) was analysed as an indicator of RVH. The lungs were then immersed in 4% paraformaldehyde overnight and wrapped with wax blocks for HE staining and immunohistochemistry. The remaining lungs were frozen in a refrigerator at −80°C for subsequent experiments.

### 2.4 Histology and immunohistochemistry

Lung tissue was fixed with 4% formaldehyde overnight, then dehydrated, cleared and embedded in paraffin. The wax blocks were cut into 4 μm thick sections for H&E and immunohistochemistry staining. For immunohistochemistry, 4‐µm paraffin‐embedded tissue sections were dewaxed and hydrated. The immunohistochemistry method was based on the technique described previously.^21^ Antibody was incubated with NDUFA4L2 (Catalogue number: 16480‐1‐AP; ProteinTech). Brown indicated positive staining.

### 2.5 Cell culture

Human PASMCs (HPASMCs) and pulmonary artery endothelial cells (HPAECs) were used in vitro experimental studies. Primary HPASMCs were derived from PAs of organ donor lungs. The PAs were cut into 1‐2 mm with ophthalmic scissors, and then digested with a mixture containing 2 mg/mL collagenase, 0.5 mg/mL elastase and 1.5 mg/mL BSA for 1‐2 hours until the tissue mass became a cell mass. Then, HPASMCs were cultured in SmGM‐2 BulletKit medium (Lonza) containing 10% FBS and placed in a 5% CO_2_ incubator at 37°C for 1 week.^22^ The purity of HPASMC was identified by a specific monoclonal antibody against smooth muscle α‐actin. Passages 2‐5 were used for the next experimental studies. HPAECs purchased from Wuxi PUHE biomedical technologies, LTD. INC and maintained in HPAECs complete medium containing 10% (volume/volume) heat‐inactivated foetal bovine serum (FBS; Gibco), 20 ng/mL VEGF, 10 ng/mL bFGF, 10 ng/mL EGF, 10 U/mL penicillin and 10 μg/mL streptomycin. Cells were incubated at 37°C in a humidified 5% CO_2_ incubator. Cells under hypoxic conditions were performed at 37°C, 92% N_2_‐5% CO_2_‐3% O_2_ mixed incubator.

### 2.6 Microarray analysis of mRNA expression

RNAs were extracted and quantified from HPASMCs according to our previously published protocol.^19^ The microarray analysis work was carried out by OE Biotech. Co., Ltd.

### 2.7 Transfection

The sequence of siRNA against NDUFA4L2 and negative control (NC) were synthesized from GenePharma (Shanghai GenePharma Co). The target sequences for using in transfection were follows: si‐h‐NDUFA4L2, sense: 5′‐CCCGCUUCUACCGGCAGAUTT‐3′; anti‐sense: 5′‐ AUCUGCCGGUAGAAGCGGGTT‐3′. si‐h‐5‐LO, sense: 5′‐CCAAAUGCCACAAGGAUUUTT‐3′; anti‐sense: 5′‐AAAUCCUUGUGGCAUUUGGCA‐3′. si‐h‐NC, sense: 5′‐ UUCUCCGAACGUGUCACGUTT‐3′; anti‐sense: ACGUGACACGUUCGGAGAATT‐3′. HIF‐1α siRNA (h) was obtained from Santa Cruz Biotechnology. The NDUFA4L2 overexpression plasmid was constructed with the PGL‐4 vector by GenePharma. HPASMCs transfection steps were performed according to the manufacturer's instructions. After transfection for 24 hours, cells were harvested for experimental analysis such as CCK‐8, EDU and Western blotting.

### 2.8 Cell cycle analysis

We used PI single staining method to detect cell cycle. Before staining, cells were washed the fixative solution with PBS (if necessary, filter the cell suspension once with a 200 mesh sieve). Hundred microliter RNase A was added to the cells at 37°C for 30 minutes. Finally, cells were added with 400 μL PI dark room to protect from light for 10 minutes. DNA fluorescence measurements were analysed using BD flow cytometry.

### 2.9 Cell Counting Kit‐8 (CCK‐8) assay

The HPASMCs were cultured in a 96‐well plate, and then cells were starved for 24 hours after growing to about 60%. The cells were transfected with Nor‐siNC, Hyp‐siNC, Hyp‐si‐NDUFA4L2 or Hyp‐si‐5‐LO. After incubation for 24 hours at 37°C, cell proliferation was detected with CCK8 kit (Dojin Laboratories, catalog no: CK04) according to the manufacturer's instruction. CCK‐8 solution was added to each well of the plate for 10 μL. The plate was incubated for 1‐4 hours in the incubator. The absorbance was measured at 450 nm using a microplate reader.

### 2.10 5‐ethynyl‐20‐deoxyuridine (EDU) staining

EdU staining was analysed using a kFluor555 Click‐It EDU Kit according to the manufacturer's protocol (KeyGen Biotech). HPASMCs were seeded into 96‐well plates at a density of 5 × 10^3^ cells/well followed by incubation for 24 hours in serum‐free DMEM. After the cells were transfected with Nor‐siNC, Hyp‐siNC, Hyp‐si‐NDUFA4L2 or Hyp‐si‐5‐LO, HPASMCs were treated with 10 μM EdU for 4 hours at 37°C. Then cells were fixed with 4% paraformaldehyde for 30 minutes at room temperature and treated with 0.5% Triton X‐100 in PBS for 20 minutes to permeabilize cells according to the manufacturer's instruction.

### 2.11 Real‐time PCR

Total RNA was extracted from HPASMCs and HPAECs using TRIzol according to the manufacturer's specifications. The yield of RNA was determined using Synergy H1 Microplate reader (BioTek), and the integrity was evaluated using agarose gel electrophoresis stained with ethidium bromide. Quantification was performed with a two‐step reaction process: reverse transcription (RT) and real‐time PCR according to the manufacturer's protocol by Ribobio co., LTD. The primer sequences were designed in the laboratory and synthesized by Sangon Biotech based on the mRNA sequences obtained from the NCBI database as follows: human NDUFA4L2, forward primer: GACAGAAAGAACAACCCGGA, reverse primer: TAGTCAGTGGAAACTGCAAGG; human β‐actin, forward primer: CATTCCAAATATGAGATGCGTT, reverse primer: TACACGAAAGCAATGCTATCAC; Rat NDUFA4L2, forward primer: TTTTGCTTTCTGCTTACACAGG, reverse primer: TCAGACACGATCAACACGTAG; Rat β‐actin, forward primer: AGGCCCCTCTGAACCCTAAG, reverse primer: CCAGAGGCATACAGGGACAAC.

### 2.12 Western blotting

The PAs homogenate tissue and collected HPASMCs and HPAECs were dissolved in proteolytic buffer for 30 minutes. After 30 minutes, the lysed PAs homogenate tissue and cell proteins were centrifuged at 13 500 *g* for 15 minutes, and then the protein concentration was measured using a BCA kit. Thirty micrograms of cell lysate from each sample were used for SDS‐PAGE (Bio‐Rad Laboratories), and Western blotting were analysed according to the protocol as described previously.^23^ The chosen antibodies included anti‐NDUFA4L2 (Catalogue number: 16480‐1‐AP; ProteinTech), anti‐HIF1α (Catalogue number: AF1009; Affinity), anti‐PCNA (Catalogue number: 10205‐2‐AP; ProteinTech), anti‐cyclin A (Catalogue number: 13295‐1‐AP; ProteinTech), anti‐cyclin E (Catalogue number: 11554‐1‐AP; ProteinTech), anti‐ERK (Catalogue number: 16443‐1‐AP; ProteinTech), anti‐p‐ERK (Catalogue number: AF1015; Affinity), anti‐5‐LO (Catalogue number: AF4699; Affinity), anti‐p‐5‐LO (Catalogue number: AF8359; Affinity), anti‐p38 (Catalogue number: 14064‐1‐AP; ProteinTech), anti‐p‐p38 (Catalogue number: 4511T; Cell Signaling), anti‐JNK (Catalogue number: 51151‐1‐AP; ProteinTech) and anti‐p‐JNK (Catalogue number: 4668S; Cell Signaling) et al. After extensive washes membranes with TBS‐T, the ECL luminescent solution is added for exposure and development.

### 2.13 4‐HNE, malondialdehyde assays

4‐HNE of plasma and HPASMCs were assessed using a ELISA Kit (BioVision, E4645‐100). HPASMCs MDA was analysed with a Lipid Peroxidation (MDA) colorimetric/fluorometric assay kit (BioVision, K739‐100). All experiments were performed according to the manufacturer's instructions.

### 2.14 Determination of mitochondrial ROS production

Cells were pre‐treated with different reagents for 24 hours. The culture medium was then removed, and ROS detection reagents DCFH‐DA (10 μM) or Rosup (5 mg/mL) (Beyotime Biotechnology) or MitoSOX Red (5 mM) (Yeasen Biotech Co., Ltd) were applied according the manufacturer's instructions. An Olympus IX73 fluorescence microscopy was used for acquisition of fluorescent images.

### 2.15 Oxygen consumption

The HPASMCs were seeded in a 96‐well culture plate and treated with hypoxia and hypoxia + siNDUFA4L2. Oxygen consumption was analysed by extracellular oxygen consumption assay kit according to the manufacturer's instructions (Abcam, ab197243).

### 2.16 Complex I activity

The HPASMCs were seeded in a 96‐well plate at a density 20 000 cells per well before the initiation of the experiment. The activity of Complex I was analysed using the Complex I Enzyme Activity Assay Kit according to the manufacturer's instructions (Abcam, ab109721).

### 2.17 Statistical analysis

The data are expressed as mean ± SEM of at least three independent experiments. One‐way ANOVA or Student's *t* test was used to determine the significance of differences between the means of different groups, followed by a Bonferroni test using the Prism software package (version GraphPad Prism 5.0). A *P* value <.05 was considered statistically significant.

## 3 RESULTS

### 3.1 mRNA expression profile in hypoxic human pulmonary artery smooth muscle cells

**Figure 1:**
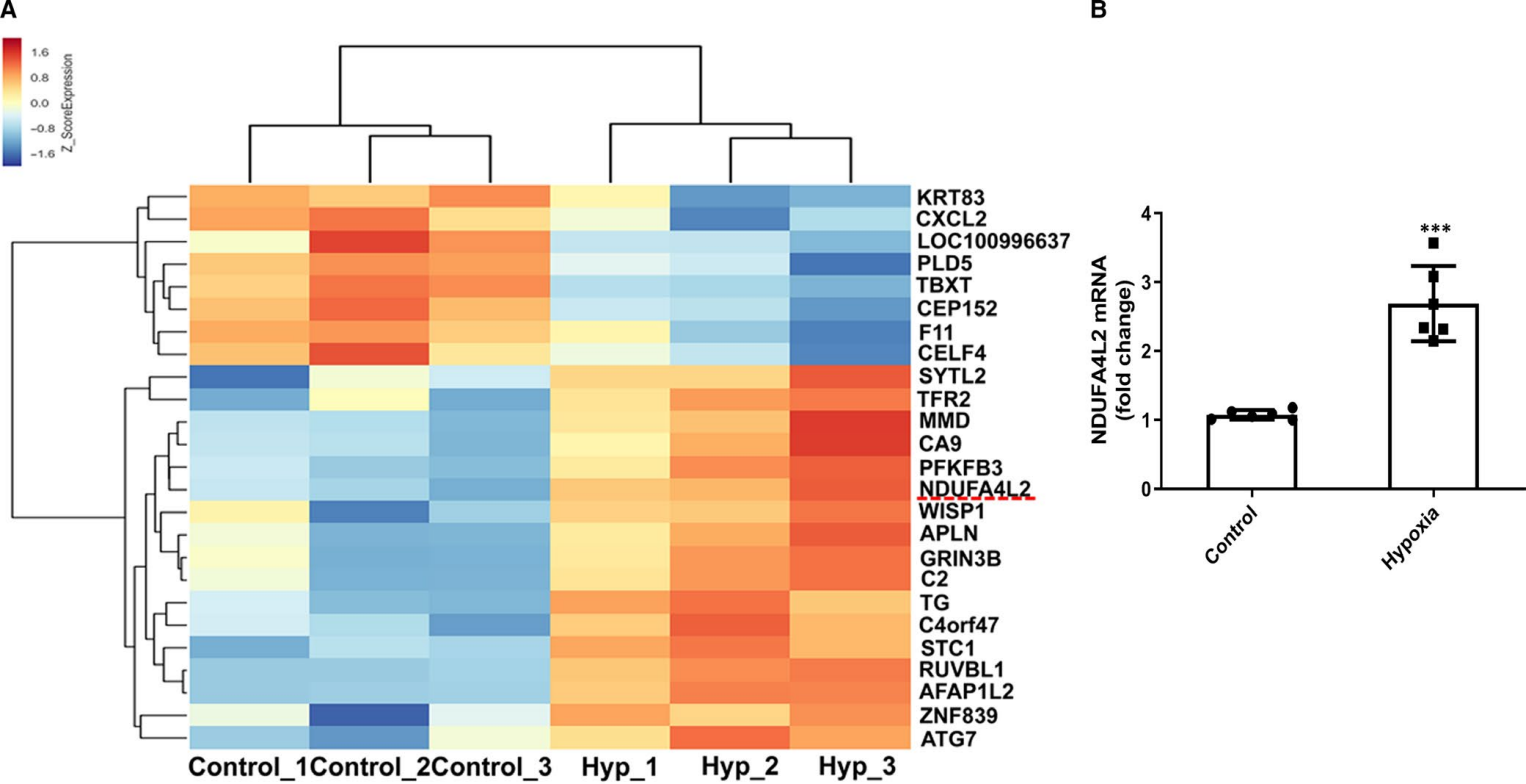
Analysis of mRNA expression profiles of normoxia and hypoxia in HPASMCs. A, Heat map of the up‐regulated and down‐regulated genes which were related to hypoxic PASMC proliferation and analysed by RNA sequencing (n = 3); (B) Real‐time PCR was used to detect mitochondrial NDUFA4L2 mRNA expression in normal and hypoxic HPASMCs (n = 6). Hyp, hypoxia. ***P < .001 vs control. All of the values are denoted as mean ± SEM

In order to find related factors that promote cell proliferation and trigger vascular remodelling in PASMCs, we compared mRNA expression profiles in normal and hypoxic HPASMCs with mRNA chips in a recognized model of hypoxic proliferation of PASMCs. We found that 17 mRNAs were significantly up‐regulated and 8 mRNAs were significantly down‐regulated with statistical differences. In order to find factors related to PASMCs proliferation, we performed GO and pathway analysis on the 25 mRNAs. Results found that NDUFA4L2 was a candidate gene for us because of its large multiples (≥8 times) (Table 1), and its results verified in subsequent in vitro and in vivo models and the chip results are reproducible (Figure 1A,B).

**Table 1:** The up‐regulated and down‐regulated expression of mRNAs in control and hypoxia‐induced HPASMC proliferation, FC (abs)>2, a total of 25

mRNA name	Regulation direction	Fold change (log_2_)	*P* value	Chromosome	Strand
APLN	Up	12.24	.02	X	−
C4orf47	Up	10.02	.001	Chr4	+
STC1	Up	8.59	.0007	Chr8	−
NDUFA4L2	Up	8.56	.003	Chr12	−
AFAP1L2	Up	7.31	.0003	Chr10	−
TG	Up	5.64	.002	Chr8	+
RUVBL1	Up	5.50	.0003	Chr3	−
C2	Up	5.41	.014	Chr6	+
CA9	Up	5.19	.019	Chr9	+
WISP1	Up	5.16	.04	Chr8	+
TFR2	Up	5.11	.02	Chr7	−
GRIN3B	Up	5.00	.02	Chr19	+
PFKFB3	Up	3.61	.006	Chr10	+
SYTL2	Up	2.18	.02	Chr11	−
ATG7	Up	2.11	.003	Chr3	+
MMD	Up	2.00	.01	Chr3	−
ZNF839	Up	2.00	.03	Chr14	+
F11	Down	4.07	.03	chr4	+
LOC100996637	Down	7.80	.03	chr2	+
TBXT	Down	5.90	.001	Chr6	−
CELF4	Down	5.14	.03	chr18	−
CXCL2	Down	4.74	.02	Chr4	−
CEP152	Down	4.71	.04	Chr15	−
PLD5	Down	4.64	.02	Chr1	−
KRT83	Down	4.17	.02	Chr12	−

### 3.2 Expression of NDUFA4L2 in the PAs of PAH patients and hypoxic PAH model rats

**Figure 2:**
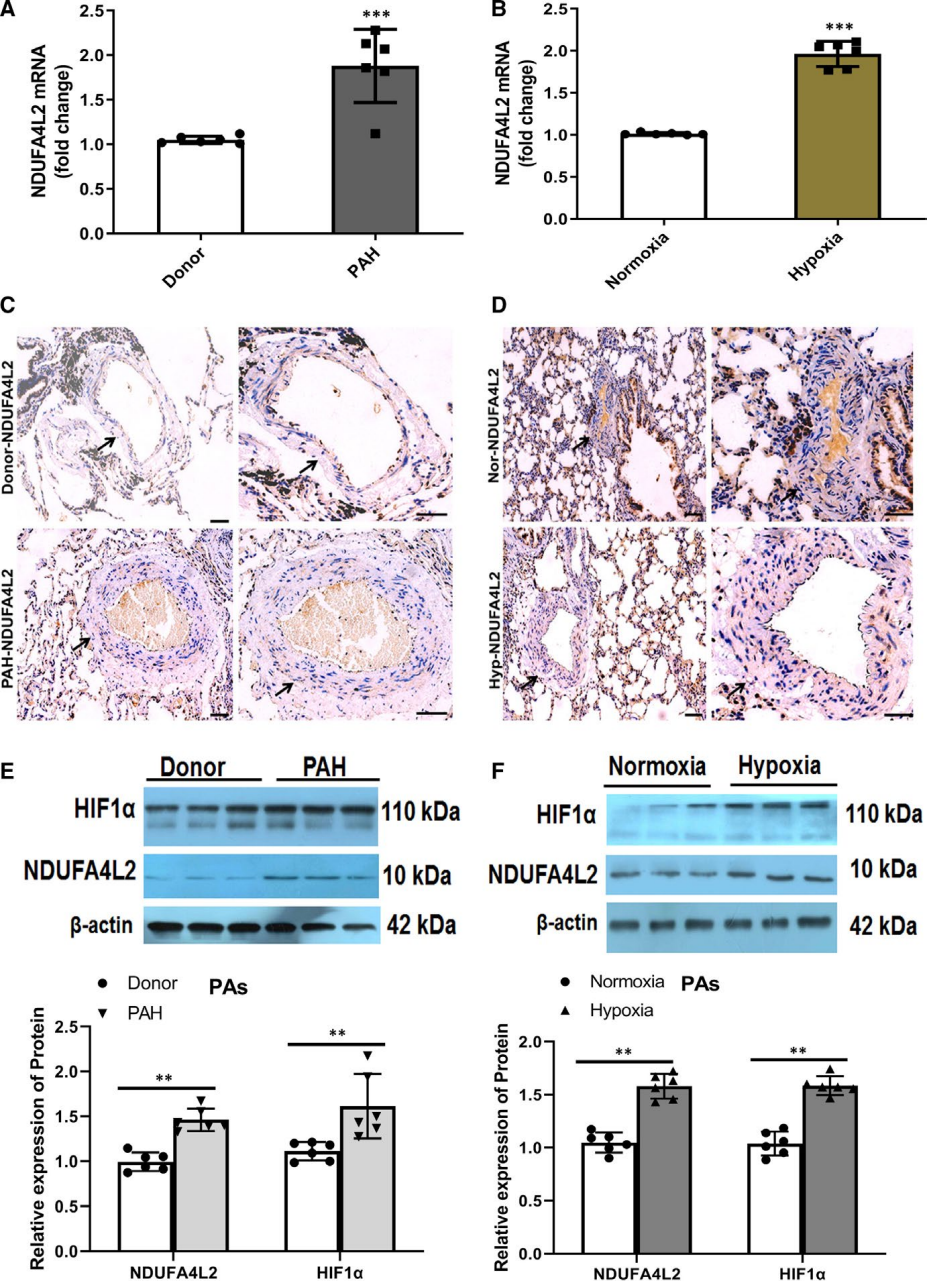
NDUFA4L2 expression was increased in the PAs of PAH patients and hypoxic PAH model rats. A,B, NDUFA4L2 mRNA expression in the PAs of PAH patients and hypoxic PAH model rats was tested by real‐time PCR; (C,D) NDUFA4L2 was immunostained in sections of PAs from PAH patients and hypoxic PAH model rats; (E,F) Western blotting analysed NDUFA4L2 and HIF1α protein expression in the PAs of PAH patients and hypoxic PAH model rats. Scale bar = 100 μm. **P < .01, ***P < .001 vs donor or normoxia group. n = 6. All of the values are denoted as mean ± SEM

Our RNA microarray results and real‐time PCR experiments have confirmed that the expression of NDUFA4L2 was increased in hypoxia HPASMCs. Next, we further verified whether the expression of NDUFA4L2 also increased in the PAs of PAH patients and hypoxic PAH model rats. The RVSP, the ratio of the weights of the free wall of the RV to the weight of wall of left ventricle plus septum (RV/LV + S), mean pulmonary artery pressure and vascular morphological characteristics were measured and analysed in hypoxic PAH model rats and PAH patients as our previously published articles.^22^, ^24^ Our results showed that both mRNA and protein levels of NDUFA4L2 were increased in the PAs of patients with PAH and in model of hypoxic rats (Figure 2A,B, E,F). Immunohistochemical experiments confirmed that NDUFA4L2 was expressed in both the medial and intimal membranes, and the median membrane was more pronounced (Figure 2C,D). Because NDUFA4L2 was a direct target of HIF‐1α, we also detected the expression of HIF‐1α. We found that HIF‐1α expression also increased in the PAs of PAH patients and hypoxic PAH model rats (Figure 2E,F).

### 3.3 Expression of NDUFA4L2 in HPASMCs and HPAECs

**Figure 3:**
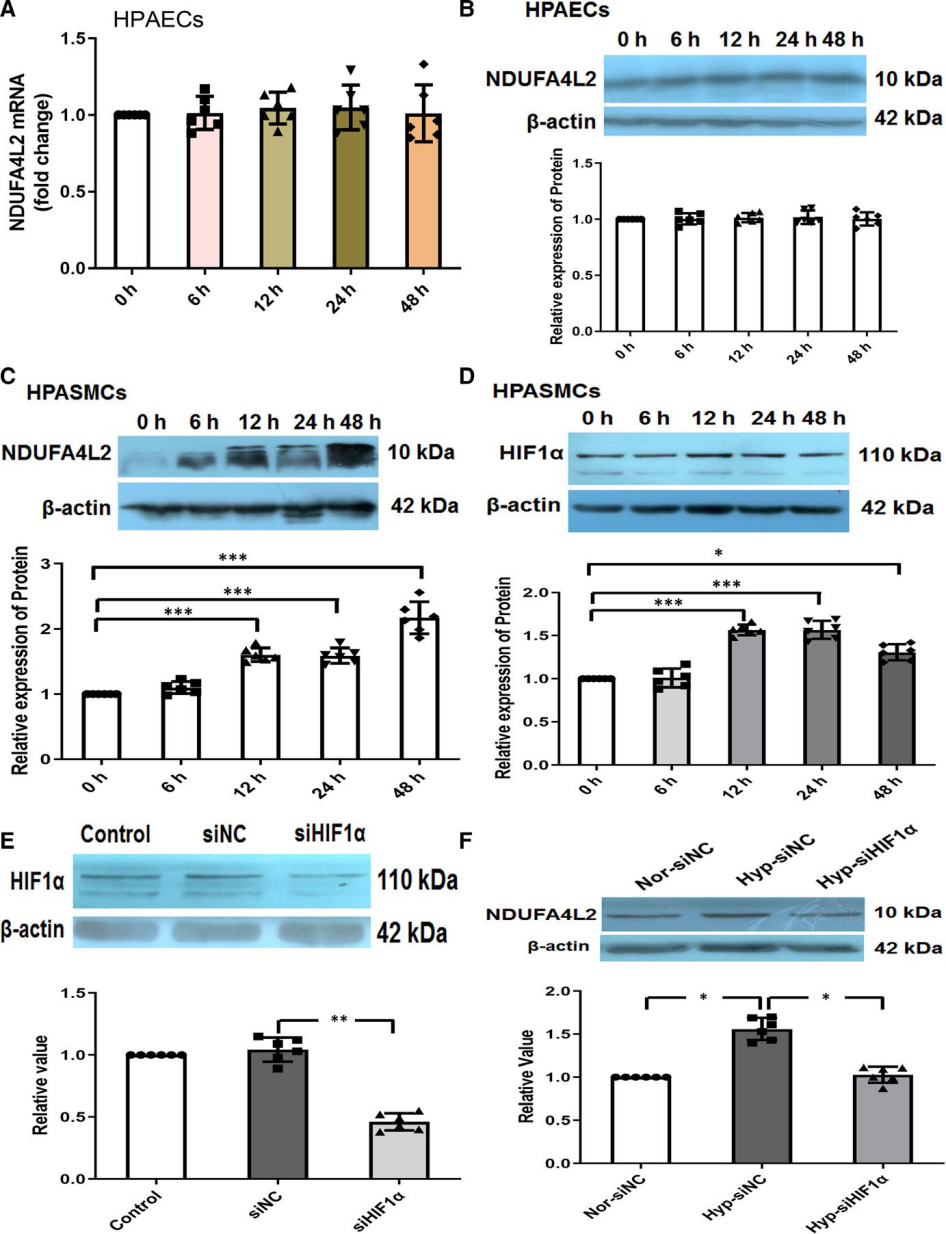
Expression of NDUFA4L2 in hypoxic HPASMCs and HPAECs. A, Real‐time PCR analysed NDUFA4L2 mRNA expression in HPAECs at different time‐points of hypoxia; (B) Western blotting analysed NDUFA4L2 protein expression in HPAECs at different time‐points of hypoxia; (C) Western blotting analysed NDUFA4L2 protein expression in HPASMCs at different time‐points of hypoxia; (D) Western blotting analysed HIF1α protein expression in HPASMCs at different time‐points of hypoxia; (E)The efficiency and specificity of siRNA directed against HIF1α (si‐HIF1α); (F) After interference with HIF1α, Western blotting was used to detect the expression of NDUFA4L2 in hypoxic HPASMCs. NC, negative control; Nor, normoxia; Hyp, hypoxia. ^*^ *P* < .05, ^**^ *P* < .01, ^***^ *P* < .001. n = 6. All of the values are denoted as mean ± SEM

Pulmonary vascular remodelling is a significant feature of PAH formation. After the pulmonary artery is subjected to various injuries or hypoxia and other stimuli, the tissue structure and function of the vascular wall undergo pathological changes, mainly including the two pathological processes of pulmonary angiogenesis and pulmonary smooth muscle thickening. Among them, pulmonary angiogenesis is caused by pulmonary artery intimal injury, and PAECs proliferation and migration, while pulmonary artery thickening is mainly caused by apoptosis inhibition and excessive proliferation of PASMCs. Therefore, it is necessary to detect the expression of NDUFA4L2 in PAECs and PASMCs in vitro. We treated the HPAECs and HPASMCs with hypoxia at different time‐points. Real‐time PCR and Western blotting experiments detected the expression of NDUFA4L2 in HPAECs, and the results showed that the expression of NDUFA4L2 did not change at different time‐points of hypoxia both mRNA and protein levels (Figure 3A,B). In contrast, the expression of NDUFA4L2 in smooth muscle cells increased with the duration of hypoxia (Figure 3C,D). Mitochondrial NDUFA4L2 has been reported as a downstream target gene of HIF‐1α. HIF‐1α induced ETC complex I subunit‐NDUFA4L2, to reduce complex I activity and ROS production.^25^ Consistent with previous reports, we found that HIF‐1α expression also showed a time‐dependent increase at different time‐points of hypoxia. To investigate whether NDUFA4L2 expression was mediated by HIF‐1α under hypoxia, we generated HPASMCs that stably expressed siRNA against HIF1a. The hypoxia‐induced NDUFA4L2 expression was markedly abolished when HIF1a was knocked down (Figure 3E,F).

### 3.4 NDUFA4L2 silencing ameliorates hypoxia‐induced PAH in rat model

**Figure 4:**
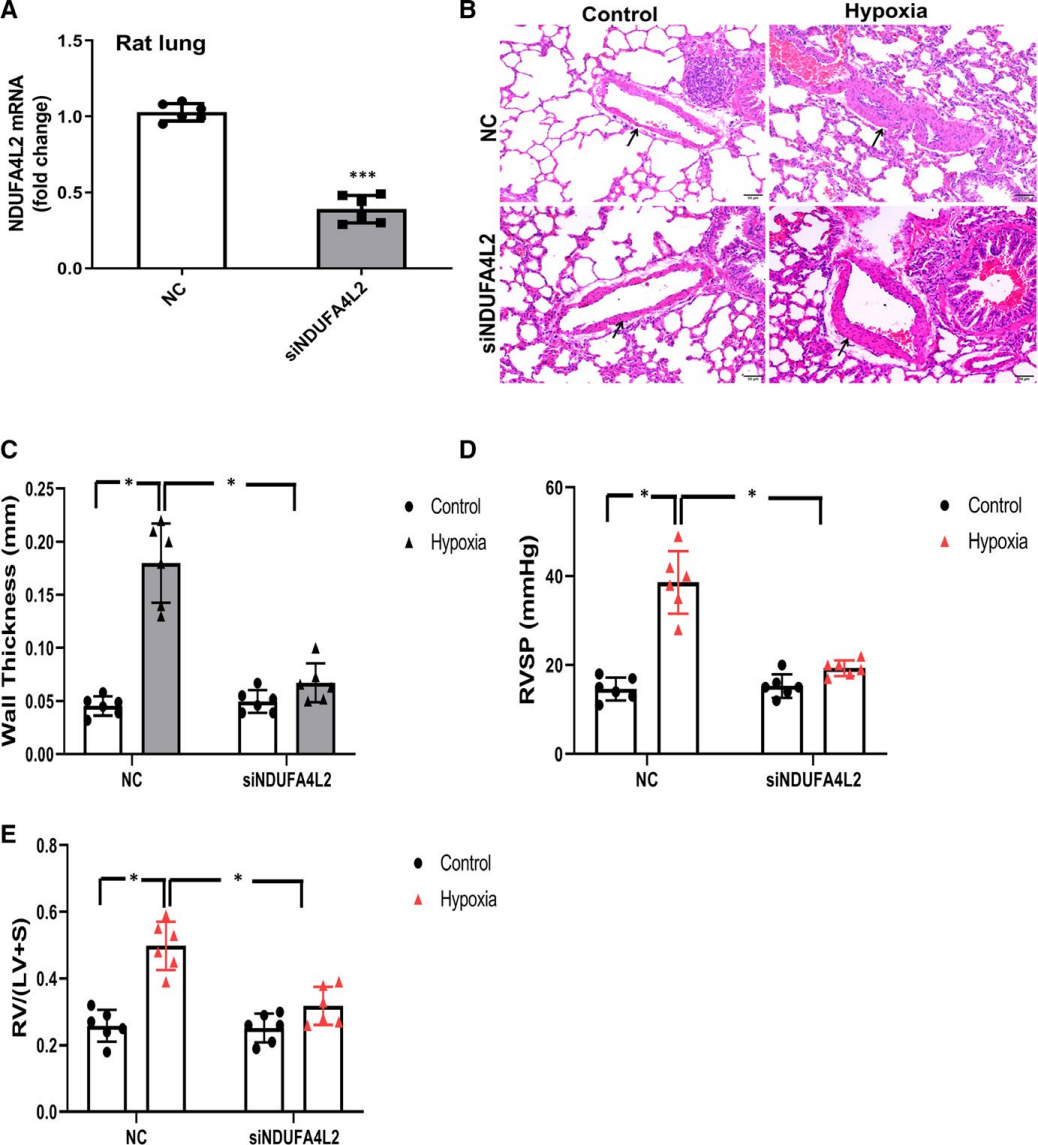
NDUFA4L2 silencing ameliorated hypoxia‐induced PAH in rat model. A, After interference with NDUFA4L2, real‐time PCR was used to detect the expression of NDUFA4L2 on rat lung; (B,C) Hypoxia‐induced thickening of pulmonary arterial wall was partially reversed by NDUFA4L2 silencing; (D) RVSP was measured in NDUFA4L2 silencing rats exposed to hypoxia; (E) Right ventricular hypertrophy was observed in hypoxia‐induced PAH, which was reversed by silencing of NDUFA4L2. NC, negative control; RVSP, right ventricular systolic pressure; RV/LV + S, right ventricle/left ventricle + septum. *P < .05, ***P < .001 vs NC, Scale bar = 50 μm. n = 6. All of the values are denoted as mean ± SEM

To understand the role of NDUFA4L2 in hypoxia‐induced PAH, Wistar rats were randomly divided into four groups (n = 6): NC_control_, NC_hypoxia,_ siNDUFA4L2_control,_ siNDUFA4L2_hypoxia_. Real‐time PCR experiment was used to examine the interference efficiency of siNDUFA4L2 on rat lung (Figure 4A). To examine that NDUFA4L2 was involved in the remodelling of pulmonary arterial wall, we first compared the thickness of PA between NC_control_ and NC_hypoxia_ group. We found significant increase of pulmonary arterial wall thickness in NC_hypoxia_ group compared with NC_control_ group, but NDUFA4L2 silencing effectively inhibited the thickening of PAs under hypoxia (Figure 4B,C). In addition, hypoxia significantly increased RVSP in NC treatment, knockdown of NDUFA4L2 led to significant decreases in RVSP in siNDUFA4L2 hypoxia group compared with NC hypoxia group (Figure 4D). Then, we observed obvious hypertrophy of RV in NC hypoxia group, but NDUFA4L2 silencing partially reversed RV hypertrophy. Quantitative analysis showed that RV/(LV + S) in NC_hypoxia_ significantly increased compared with NC_control_, but NDUFA4L2 silencing significantly decreased RV/(LV + S) in hypoxia condition (Figure 4E).

### 3.5 The activation of NDUFA4L2 promoted proliferation of hypoxic HPASMCs

**Figure 5:**
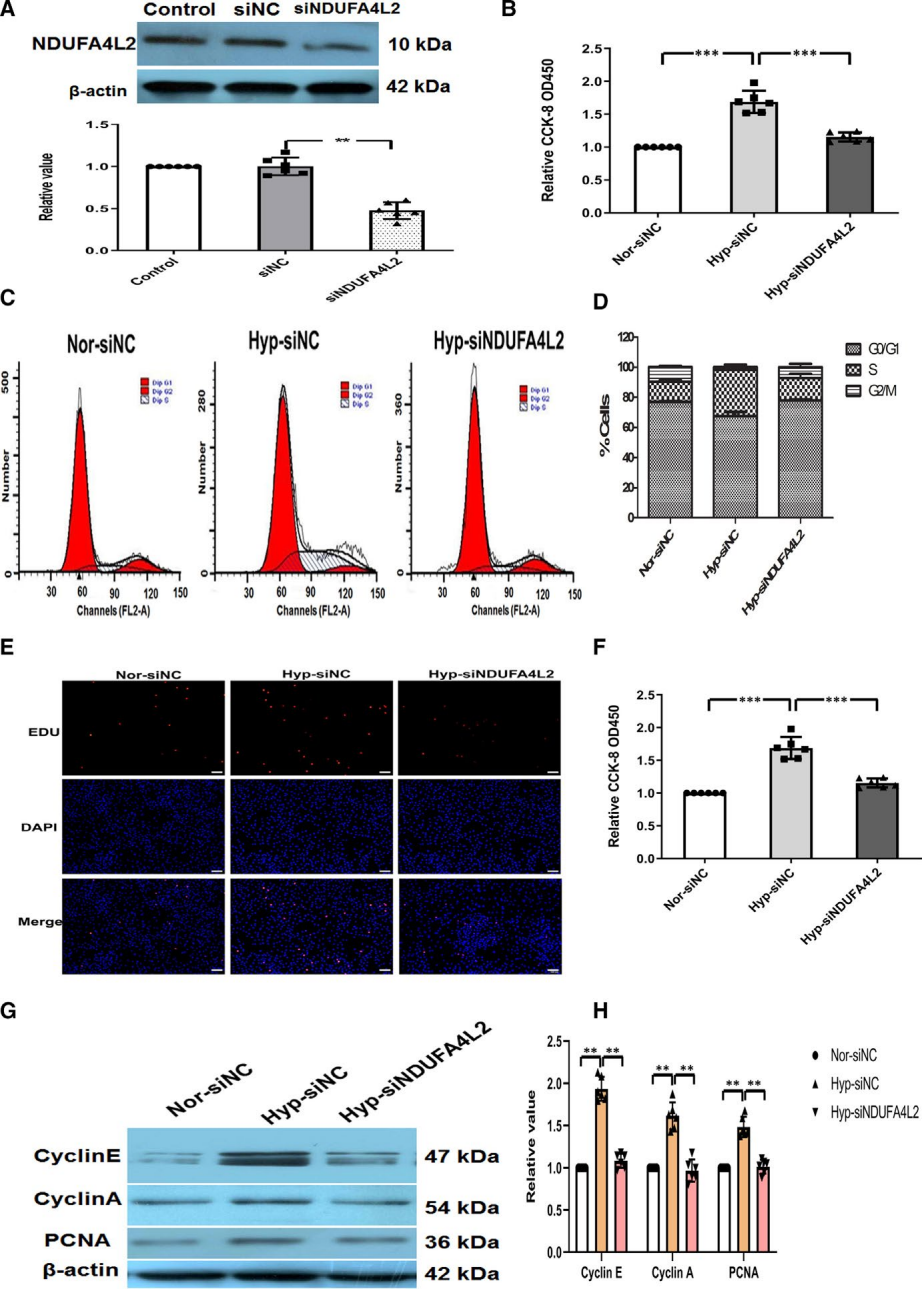
NDUFA4L2 activation promoted proliferation of hypoxic HPASMCs. A, The efficiency and specificity of siRNA directed against NDUFA4L2 (si‐NDUFA4L2); (B) CCK‐8 was analysed the proliferation of HPASMCs under normoxia, hypoxia and hypoxia + si‐NDUFA4L2; (C) Cell cycles was analysed the proliferation of HPASMCs after knockdown of NDUFA4L2 in hypoxia condition; (D) The histogram showed the percentage of cells in the G_0_/G_1_, S and G_2_/M phases; (E) EdU staining was analysed the proliferation of NDUFA4L2 in hypoxic HPASMCs; (F) Quantitative analysis of EdU‐positive staining; (G) Western blotting examined the expression of PCNA and cycle‐related protein after knockdown of NDUFA4L2 in hypoxia condition; (H) The histogram showed the expression results of cycle‐related protein. NC, negative control; Nor, normoxia; Hyp, hypoxia. Scale bar = 50 μm. ^**^ *P* < .01, ^***^ *P* < .001. n = 6. All of the values are denoted as mean ± SEM

To examine the effect of NDUFA4L2 on the proliferation of hypoxic HPASMCs, we knocked out the gene NDUFA4L2 and treated the HPASMCs with hypoxia. We designed siRNA to inhibit NDUFA4L2 expression. The efficiency of siRNA transfection was tested and confirmed by Western blotting (Figure 5A). CCK‐8 assay indicated that the proliferation of PASMCs was inhibited in the hypoxia + si‐NDUFA4L2 group compared with the hypoxia group (Figure 5B). To understand whether NDUFA4L2 affected the cell cycle progression, the number of cells in the different cell cycle phases was detected by flow cytometry. The results showed that hypoxia increased the percentage of cells in S + G_2_/M phase. NDUFA4L2 knockdown suppressed the cell cycle progression and made more HPASMCs arrested at the G_0_/G_1_ phase in hypoxia condition (Figure 5C,D). As shown in Figure 5E,F, EdU staining assay showed higher fraction of proliferating cells in the hypoxia groups cells compared with NC siRNA transfected cells. This effect was reversed in the presence of NDUFA4L2 siRNA in hypoxic condition. Because PCNA, cyclin A and cyclin E played important roles in the cycle process, we analysed the protein expression of PCNA, cyclin A and cyclin E in HPASMC. Our results indicated that hypoxia enhanced the expression of PCNA, cyclin A and cyclin E, but the effect was decreased after NDUFA4L2 silencing (Figure 5G,H). These data indicated that NDUFA4L2 knockdown decreased hypoxia‐induced proliferation in HPASMCs.

### 3.6 Hypoxia increases the production of 4‐HNE, MDA and NDUFA4L2 is involved in the hypoxia‐induced increase in ROS production and decrease in complex I activity and oxygen consumption during hypoxia

**Figure 6:**
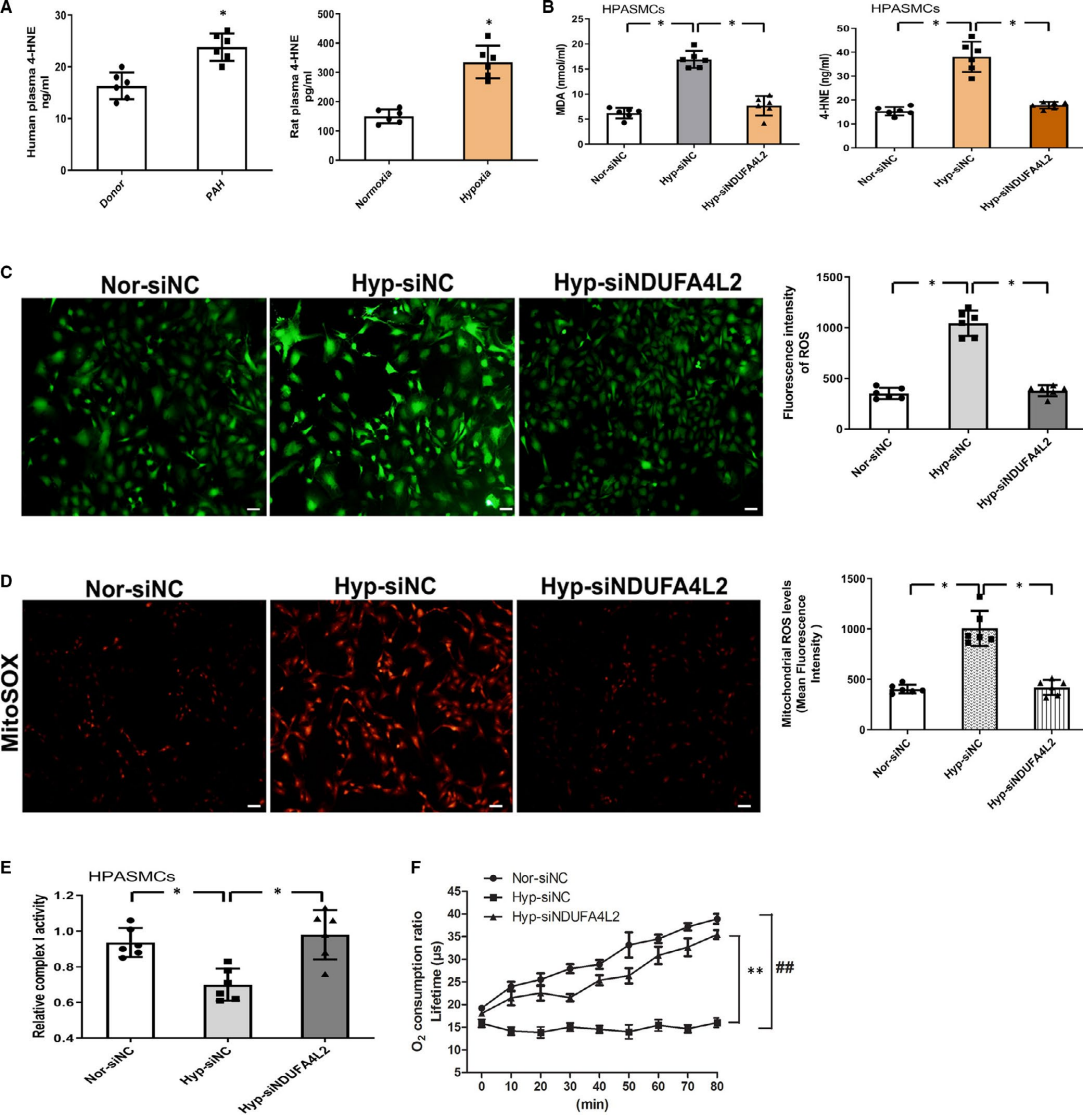
Lipid peroxidation was increased in pulmonary arterial hypertension and NDUFA4L2 silencing‐mediated inhibition of hypoxia‐induced oxidative stress and mitochondrial dysfunction. A, Plasma 4‐HNE levels were determined by ELISA (n = 6); (B) After knockdown of NDUFA4L2 in HPASMCs in vitro, ELISA was detected the expression of lipid peroxidation metabolites 4‐HNE and MDA in different groups of Nor‐siNC, Hyp‐siNC and Hyp‐siNDUFA4L2 (n = 6); (C) Immunofluorescence was analysed the ROS release after interference with siNDUFA4L2 in HPASMCs (n = 6); (D) Representative images of six independent experiments showing MitoSOX intensity as a measure of mitochondrial superoxide levels (n = 6); ROS production was increased in hypoxia‐treated NC, which was reversed by silencing of NDUFA4L2; (E) Complex I activity was measured in HPASMCs transfected with siNC or NDUFA4L2 siRNA, then exposed to normoxic or hypoxic (3% O_2_) conditions for 24 h (n = 6); (F) Oxygen consumption ratio in siNC or NDUFA4L2 siRNA, then exposed to normoxic or hypoxic (3% O_2_) conditions for 24 h (n = 3). NC, negative control; Nor, normoxia; Hyp, hypoxia. *P < .05 vs donor or normoxia group; ^*^ *P* < .05, ^**^ *P* < .01, ^##^ *P* < .01, Scale bar = 100 μm. All of the values are denoted as mean ± SEM

Hypoxia causes mitochondria to produce excessive ROS to attack and promote oxidation of lipid, especially polyunsaturated fatty acids, a process called lipid peroxidation. Lipid peroxidation produces a variety of oxidation products, of which aldehyde is the main end product. Among the lipid peroxidation products, 4‐HNE is considered the most toxic aldehyde and MDA appears to be the most mutagenic aldehyde. To evaluate lipid peroxidation in PAH, levels of 4‐HNE and MDA were examined. 4‐HNE level was significantly increased in plasma of both PAH patients and hypoxic rats compared with that in donor and normoxic rats (Figure 6A). Consistent with the above results, hypoxic PASMCs also increased the levels of 4‐HNE and MDA, but this effect was disappeared after NDUFA4L2 silencing (Figure 6B). In vitro and in vivo, data showed that PAH increased lipid peroxidation. As one of the main end products of lipid peroxidation, 4‐HNE is positively correlated with oxidative stress. We verify the effect of NDUFA4L2 on oxidative stress. Hypoxia‐treated HPASMCs were incubated with DCFH‐DA and mitochondrial superoxide indicator MitoSOX for ROS detection, and changes in oxidative stress levels were determined using fluorescent microscopic imaging. The levels of oxidative stress were significantly higher in HPASMCs exposed to hypoxia for 24 hours compared with that in untreated cells, indicating that hypoxia enhanced oxidative stress, and the effect was reversed after NDUFA4L2 silencing (Figure 6C,D). Furthermore, in order to examine whether NDUFA4L2 was involved in the regulation of mitochondrial activity in hypoxia, complex I activity (Figure 6E) and the mitochondrial oxygen consumption ratio (Figure 6F) were measured. The results showed that hypoxia decreased complex I activity and mitochondrial O_2_ consumption ratio. In contrast, NDUFA4L2 silencing treatment protected complex I activity and the mitochondrial O_2_ consumption ratio from hypoxia. These results indicated that knockdown of NDUFA4L2 protects HPASMCs from hypoxia‐induced oxidative stress and mitochondrial dysfunction.

### 3.7 p38‐5‐LO may be the downstream pathway of NDUFA4L2 inducing PAH

**Figure 7:**
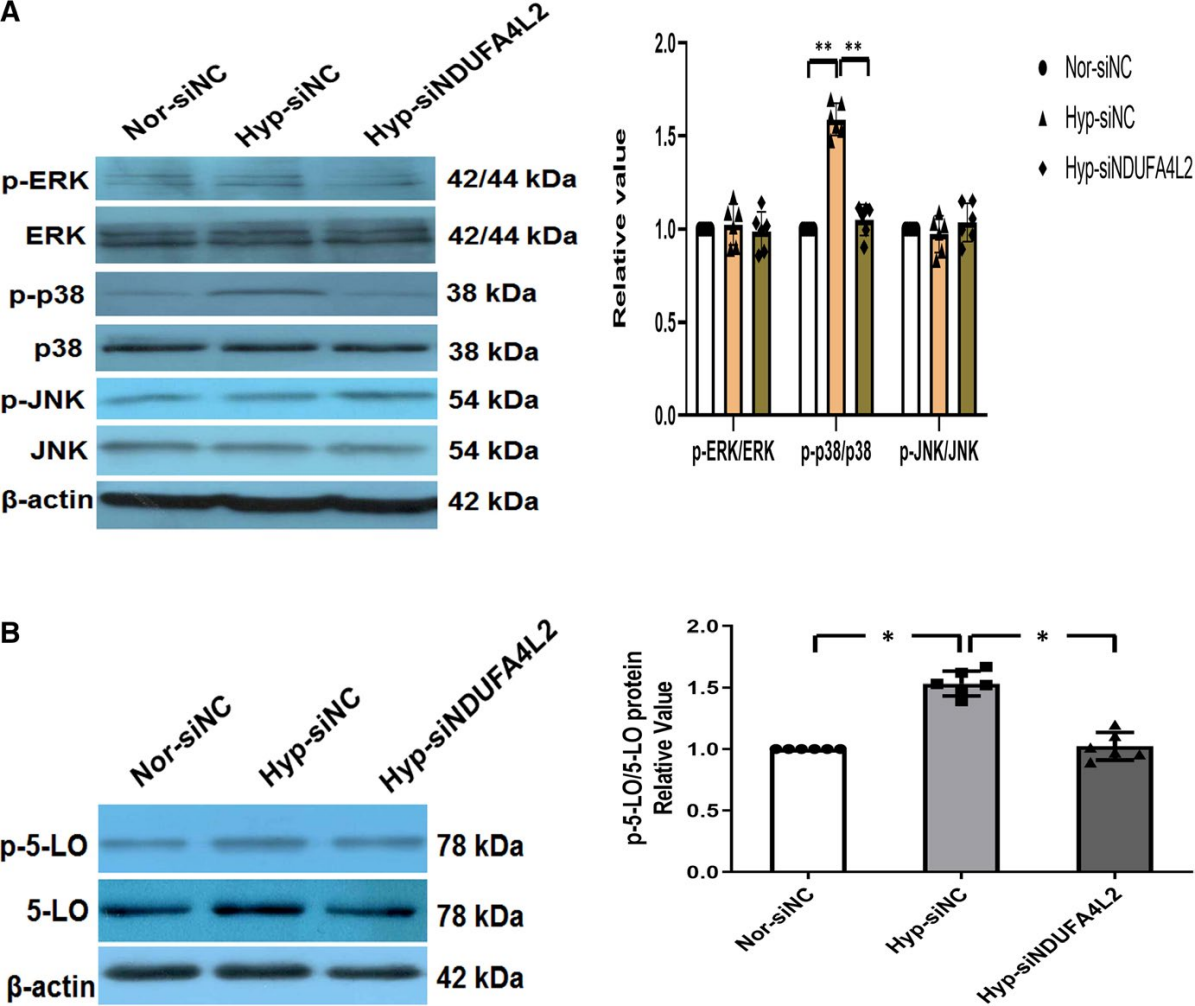
NDUFA4L2 regulated downstream p38‐5‐LO pathway may be an important mechanism for hypoxic pulmonary vascular remodelling. A, After interference with NDUFA4L2, Western blotting was used to detect the expression of p‐ERK/ERK, p‐p38/p38, p‐ERK/ERK in hypoxic HPASMCs; (B) After interference with NDUFA4L2, Western blotting was used to detect the expression of p‐5‐LO/5‐LO in hypoxic HPASMCs. NC, negative control; Nor, normoxia; Hyp, hypoxia. ^*^ *P* < .05, ^**^ *P* < .01. n = 6. All of the values are denoted as mean ± SEM

Next, we want to know how NDUFA4L2 works? Our results indicated that knockdown of NDUFA4L2 significantly down‐regulated the expression of phosphorylated p38 MAPK protein in HPASMCs hypoxic model, while the proteins at the phosphorylation sites of JNK and ERK have not changed (Figure 7A). As early as 2001, oxidative stress has been reported to activate p38.^26^ p38 activation could increase 5‐LO activity by promoting 5‐LO phosphorylation in cells.^27^ The increase in 5‐LO activity will aggravate the occurrence of PAH.^28^ To test whether 5‐LO played an important role in HPASMCs as a downstream target of NDUFA4L2, we detected the expression of 5‐LO. Expression of 5‐LO and p‐5‐LO were increased in HPASMCs under hypoxia, while knockdown of NDUFA4L2 inhibited the expression of 5‐LO and p‐5‐LO (Figure 7B). These results suggested that p38‐5‐LO may be a downstream pathway of NDUFA4L2‐induced PASMCs proliferation during PAH.

### 3.8 p38‐5‐LO as a downstream molecular signal regulated NDUFA4L2 to affect PASMCs proliferation

**Figure 8:**
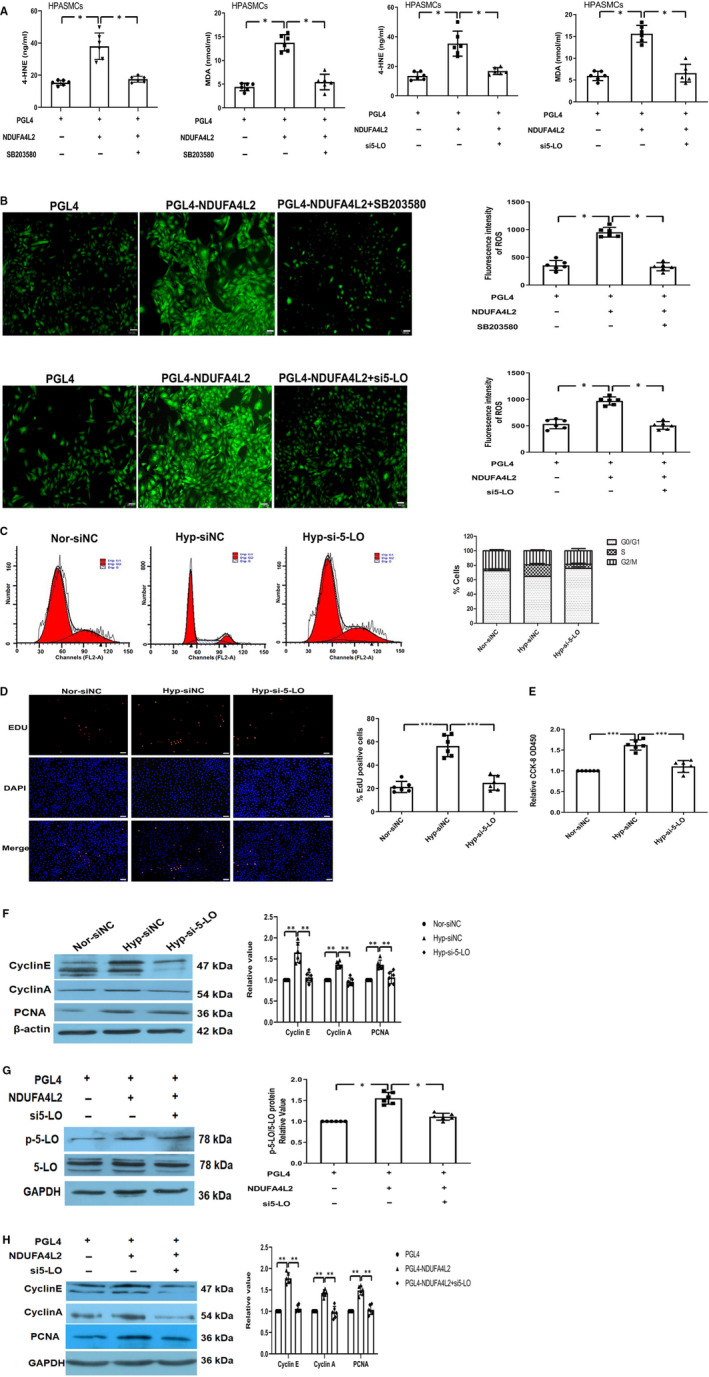
NDUFA4L2 promoted lipid oxidation and ROS production through the p38/5‐LO pathways and increased HPASMCs proliferation by 5‐LO‐dependent lipid peroxidation. A, The levels of lipid peroxidation metabolites 4‐HNE and MDA were increased after overexpression of NDUFA4L2 in HPASMCs in vitro and reversed by p38 inhibitor SB203580 or si5‐LO; (B) DCFH‐DA for ROS detection showed the ROS amounts in different experimental groups; (C) Cell cycles were analysed the proliferation of HPASMCs after knockdown of 5‐LO in hypoxia condition. The histogram showed the percentage of cells in the G_0_/G_1_, S and G_2_/M phases; (D) EdU staining was analysed the proliferation of 5‐LO‐dependent lipid peroxidation in regulation of HPASMCs proliferation in hypoxic condition; (E) CCK‐8 analysed the proliferation of HPASMCs under normoxia, hypoxia and hypoxia + si5‐LO; (F) Western blotting examined PCNA and cycle‐related protein expression after knockdown of 5‐LO in hypoxia condition; (G) The expression of p‐5‐LO/5‐LO was increased in overexpression of NDUFA4L2 in HPASMCs in vitro and reversed by 5‐LO silencing; (H) The protein expression of PCNA and cycle‐related proteins was determined by Western blot analysis. NC, negative control; Nor, normoxia; Hyp, hypoxia. Scale bar = 50 μm. ^*^ *P* < 0.05, ^**^ *P* < .01, ^***^ *P* < .001. n = 6. All of the values are denoted as mean ± SEM

To examine whether NDUFA4L2 promoted lipid oxidation and ROS production through the p38/5‐LO pathway, levels of 4‐HNE and MDA were examined. Our data showed that the levels of lipid peroxidation metabolites 4‐HNE and MDA were increased after overexpression of NDUFA4L2 in HPASMCs in vitro and reversed by p38 inhibitor SB203580 or si5‐LO (Figure 8A). DCFH‐DA for ROS detection showed that ROS production was increased after overexpressing NDUFA4L2 in HPASMCs, and the effect was reserved by SB203580 or 5‐LO silencing (Figure 8B). Consistent with previous studies, we found that 4‐HNE, as one of the major end products of lipid peroxidation, could promote ROS production and pulmonary artery smooth muscle proliferation.^12^ However, the role of 5‐LO in regulating PASMC function has not been demonstrated. Next, 5‐LO‐dependent lipid peroxidation regulating HPASMCs proliferation was evaluated. To address this issue, we transfected cultured HPASMCs with 5‐LO silencing under hypoxia or NDUFA4L2 activation. The effect of 5‐LO on cell cycle progression showed that knockdown of 5‐LO reduced the percentage of G_2_/M + S under hypoxic conditions, accompanied by an increase in cells in the G_0_/G_1_ phase (Figure 8C). In addition, the percentage of EdU‐positive cells was increased under hypoxia, which was decreased by 5‐LO silencing (Figure 8D). Similarly, CCK‐8 assay indicated that the proliferation of PASMCs was increased in HPASMCs under hypoxic and the effect was reversed after 5‐LO silencing (Figure 8E). Then, the expression of PCNA and cell cycle‐related proteins cyclin A, and cyclin E was analysed. Our results showed that hypoxia increased the expression of PCNA, cyclin A and cyclin E, and the effect was reserved by knockdown 5‐LO (Figure 8F). Then, we directly detected the effect of 5‐LO‐dependent lipid peroxidation on the proliferation of smooth muscle cells by overexpression NDUFA4L2. We found overexpressing NDUFA4L2 increased the expression of p‐5‐LO/5‐LO, 5‐LO silencing inhibited the effect (Figure 8G). We then analysed PCNA and post‐translational levels of cell cycle–related proteins cyclin A and cyclin E in HPASMCs. Expression of PCNA, cyclin A and cyclin E increased significantly under overexpressing NDUFA4L2 conditions. However, these effects were reversed after 5‐LO knockdown under the same conditions (Figure 8H). These results confirmed that 5‐LO‐dependent lipid peroxidation played an important role in regulation of HPASMC proliferation.

## 4 DISCUSSION

Abnormal proliferation of PASMCs is an important pathological feature of pulmonary arterial remodelling in HPH.^29^ To find the related factors on cell proliferation and vascular remodelling in PASMCs, we compared mRNA expression profiles of normal and hypoxic HPASMCs with mRNA chips in a recognized model of hypoxia proliferation in PASMCs and further detected a novel role for the NDUFA4L2 in HPASMCs proliferation, upstream target and downstream signalling pathways. Our studies discovered that mitochondrial NDUFA4L2, a component of the ETC complex I subunit, was overexpressed in the PAs of PAH patients and hypoxic PAH model rats. NDUFA4L2 was also overexpressed in HPASMCs cultured in hypoxic conditions. We further confirmed that HIF‐1α was an upstream target of NDUFA4L2. We found that plasma levels of 4‐HNE were increased both in PAH patients and hypoxic PAH model rats. Knockdown of NDUFA4L2 decreased the levels of MDA and 4‐HNE in HPASMCs under hypoxic conditions. Elevated MDA and 4‐HNE levels might be associated with excessive ROS generation and increased expression of 5‐LO in hypoxia, but this effect was blocked by combined treatment with siNDUFA4L2. These findings provided new insights into the key role of mitochondrial NDUFA4L2 in HPH, and NDUFA4L2 could be a potential pharmacological target for the therapy of HPH and other lipid peroxidation‐related diseases.

Complex I is the initiating complex for electron transfer in the respiratory chain. In addition, complex I is also the main site of ROS production in mitochondria except complex III. Among them, NDUFA4L2, as a component of the ETC complex I subunit, belongs to the complex I subunit NDUFA4 subunit family, which can fine‐tune complex I activity; thus, the mitochondria activates oxidative phosphorylation and produces ROS. NDUFA4L2, which is one of the important components of the ETC complex I subunit in mitochondria, is an important site for ROS generation and is widely involved in the regulation of biological processes. Previous research showed the expression of NDUFA4L2 was increased in multiple cancer cells, including clear cell renal cell cancer,^16^, ^30^ hepatocellular carcinoma^14^ and colorectal cancer.^17^ NDUFA4L2 knockdown has a profound antiproliferative effect in HIF1α‐positive cell lines.^18^ In addition, mitochondrial NDUFA4L2 is a downstream target gene of HIF‐1α. It can reduce oxygen consumption and complex I activity under hypoxia. NDUFA4L2 participates in hypoxia adaptation by reducing mitochondrial ROS production.^25^ This suggests that NDUFA4L2 plays a role in balancing apoptosis and proliferation by regulating intracellular ROS levels. In the pulmonary circulation, the accumulation of intracellular ROS is an important factor in the proliferation of PASMCs. Previous studies have found that hypoxia could significantly increase the expression of NDUFA4L2 in hepatocellular carcinoma and lung cancer. Further research found that NDUFA4L2 was a direct target of HIF‐1α. NDUFA4L2 transcription and response levels were reduced after HIF‐1α gene knockout or treatment with its inhibitor digoxin. In addition, during the occurrence of PAH, a large amount of literature also reported that HIF‐1α increased significantly and promoted PVR. In our study, we used a well‐known hypoxia PASMC cell proliferation model for mRNA chip expression profiling. Consistent with previous studies, the significantly up‐regulated NDUFA4L2 gene was also significantly increased in the PAs of PAH patients and hypoxic PAH model rats. The inhibition of NDUFA4L2 expression with 5′Chol + 2′‐O‐methyl (2′‐OMe) modified oligonucleotide could significantly reduce hypoxia‐induced PVR and haemodynamic increase in rat lung tissues. NDUFA4L2 inhibition may be an important method for the treatment of PAH. Increased NDUFA4L2 was accompanied by increased expression of HIF‐1α. In hypoxic‐treated HPASMCs and HPAECs, the results showed that the expression of NDUFA4L2 was increased in a time‐dependent manner in HPASMCs under hypoxic conditions, while HPAECs did not change significantly at different time‐points. HIF1α also increased in a time‐dependent manner in HPASMCs under hypoxic conditions. Similarly, the expression of NDUFA4L2 decreased after HIF1α was knocked out. These results suggested that HIF‐1α could participate in PVR by activating NDUFA4L2 expression during PAH.

Smooth muscle cell proliferation plays an important role in hypoxic PVR. Chronic hypoxia promotes the proliferation of PASMCs, leading to thickening of the pulmonary arterial wall and narrowing of the lumen, which in turn leads to increased pulmonary vascular resistance, RVH and continuous increase in pulmonary arterial pressure. Although the effect of hypoxia on the proliferation of PASMCs is controversial, more and more studies have demonstrated the accelerating effect of hypoxia in vitro. In the present study, we found that hypoxia promoted HPASMC proliferation. More importantly, NDUFA4L2 functions as a regulatory molecule in hypoxia‐induced smooth muscle cell proliferation as shown by using siNDUFA4L2.

In addition, NDUFA4L2‐mediated oxidative stress may be an important factor in the pathogenesis of PAH. NDUFA4L2 activation‐mediated ROS release in mitochondria may be a major form of pulmonary vascular regulation. Hypoxia causes excessive production of ROS that originate from mitochondria and NOXs (nicotinamide adenine dinucleotide phosphate oxidases)^10^ and attack and promote the oxidation of lipids, especially polyunsaturated fatty acids, a process called peroxidation.^11^ Lipid peroxidation produces a variety of oxidation products, with aldehydes being the major end products. Toxic aldehydes are highly reactive and interact with cellular macromolecules, including nucleic acids and proteins to produce various adducts, resulting in DNA damage and protein inactivation. Among the products of lipid peroxidation, 4‐HNE is considered the most toxic aldehyde and MDA appears to be the most mutagenic aldehyde.^11^ Lipid oxidation has been reported to participate in PASMC proliferation and exacerbate PAH development.^12^ Our study demonstrated that lipid peroxidation product 4‐HNE content was significantly increased in the plasma of PAH patients and hypoxia‐induced PAH rat models. Increased effect of 4‐HNE and MDA was inhibited after hypoxia treatment in cultured HPASMCs in vitro by siNDUFA4L2 interference.

Very intriguingly, our data showed that hypoxia decreased complex I activity and oxygen consumption via up‐regulation of NDUFA4L2. Previous study also found hypoxia‐induced NDUFA4L2 attenuated mitochondrial oxygen consumption involving inhibition of complex I activity, which limited the intracellular ROS production under low‐oxygen conditions.^25^ Paradoxically, in our study, hypoxia‐induced NDUFA4L2 reduces mitochondrial oxygen consumption, which involves the inhibition of complex I activity, and on the contrary increases the production of ROS. Although the increase or decrease of ROS production in PAH and cancer is controversial, a large number of literature reports support that increased ROS production is one of the pathogenic factors causing PH.^12^, ^31^, ^32^, ^33^, ^34^ The ROS production pathway is mainly mitochondrial, oxidase activity, peroxisome activity, endoplasmic reticulum stress, etc The lipid oxidation pathway, as one of the pathways to generate oxygen free radicals, produces a large number of oxidation products. We observed that the increase of 4‐HNE and MDA in PAH and hypoxic HPASMC may contribute to the release of ROS. Thus, ROS generation is predominantly from elevated 4‐HNE and MDA in PAH and hypoxic‐HPASMs, while 4‐HNE has been shown to be the predominant source in the hypoxic lung.^12^ Elevated 4‐HNE level might be associated with excessive ROS generation and increased expression of 5‐LO.^12^ The activity or expression of LOs in PAs is also increased by exposure to chronic hypoxia.^32^, ^35^ Consistent with previous studies, we found increased ROS generation and expression of 5‐LO in chronic hypoxic lung tissues, PAH patients’ lung tissues and hypoxic HPASMCs. Although the role of mitochondrial‐derived ROS in PAH and cancer is controversial, studies have shown that mitochondrial ROS can be increased or decreased. While mitochondrial ROS have been shown to be increased in PAH associated with aldehyde dehydrogenase 2^12^ and in PAEC exposed to ET‐1^33^ or ADMA.^34^ PAH and cancer have similar pathological characteristics of abnormal cell proliferation and apoptosis resistance. However, the pathogenesis of PAH is still very different from cancer. As our study found, the activation of lipoxygenase pathway by PH leads to an increase in ROS, which may be one of the reasons for the inconsistency. Although more work is needed to clarify the role of mitochondrial ROS in the occurrence of PAH, it is reasonable to conclude that the PAH field has reached a consensus that mitochondria play an important role in the occurrence of PAH. These results strongly support that NDUFA4L2 may be a new signal intermediary molecule that regulates PAH occurrence.

Next, we want to know how NDUFA4L2 works? Our results showed that knockdown of NDUFA4L2 significantly down‐regulated the expression of phosphorylated p38 MAPK and 5‐LO proteins, while the proteins did not change at the phosphorylation sites of JNK and ERK in hypoxic PASMC cell model. As early as 2001, studies have reported that oxidative stress activates p38 MAPK.^26^ This report suggests that p38 MAPK may be an important downstream molecule for NDUFA4L2 to mediate cell proliferation. p38 MAPK activation could increase 5‐LO activity by promoting phosphorylation of 5‐LO in cells.^27^ The increase activity of 5‐LO tends to aggravate PAH.^28^ Moreover, 5‐LO overexpression markedly accelerated the progression of PH in rats induced by monocrotaline.^36^ Increased expression of 5‐LO in primary PAH endothelial cells promoted proliferation of PAECs and triggered vascular remodelling.^37^ These reports suggested that increased 5‐LO activity could promote PAH. Therefore, p38‐5‐LO may be downstream pathway which NDUFA4L2 induces proliferation of PASMCs.

**Figure 9:**
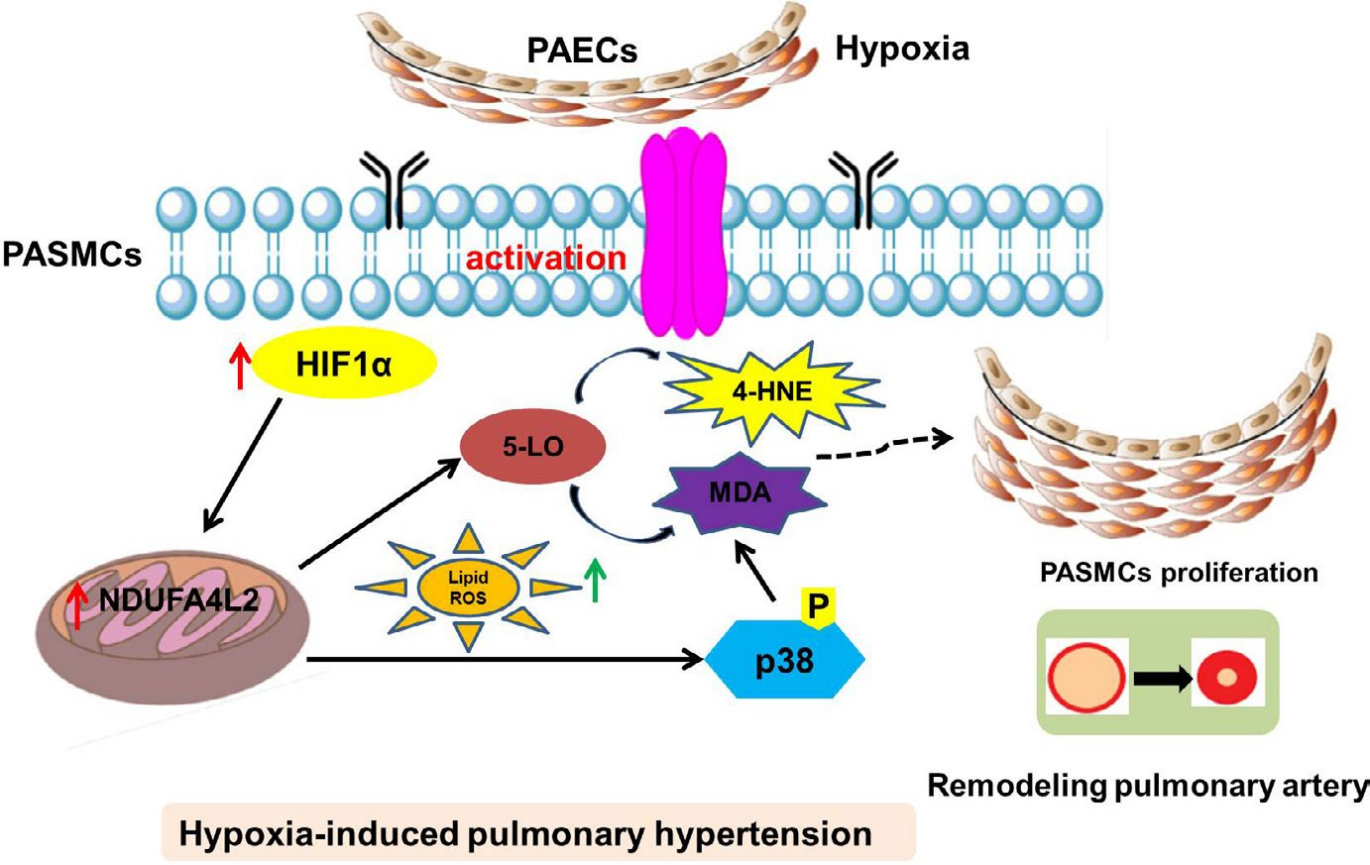
A proposed model of HIF1α‐NDUFA4L2‐p38/5‐LO axis promoting smooth muscle cell proliferation and mediating vascular remodelling. PAECs, pulmonary artery endothelial cells; PASMCs, pulmonary artery smooth muscle cells; HIF, hypoxia‐inducible factor; ROS, reactive oxygen species; NDUFA4L2, NADH dehydrogenase (ubiquinone) 1 alpha subcomplex 4 like 2; 5‐LO, 5‐lipoxygenase; 4‐HNE, 4‐hydroxynonene; MDA, malondialdehyde

In conclusion, the present study demonstrated that NDUFA4L2 played a key role in the development of HPH by activating HIF‐1α, inducing phosphorylation of p38, promoting lipid oxidation, which in turn activates 5‐LO, and subsequently enhancing PASMC proliferation (Figure 9). Furthermore, HIF‐1α‐NDUFA4L2‐p38/5‐LO could be a potential pharmacological target for the therapy of HPH and other lipid peroxidation related diseases.

## CONFLICT OF INTEREST

The authors declare no conflict of interest.

## AUTHOR CONTRIBUTIONS

**Yun Liu:** Funding acquisition (equal); Writing‐original draft (equal). **Xiaowei Nie:** Data curation (equal). **Jinquan Zhu:** Methodology (equal). **Tianyan Wang:** Methodology (equal). **Yanli Li:** Methodology (equal). **Qian Wang:** Conceptualization (equal); Resources (equal). **Zengxian Sun:** Conceptualization (equal); Resources (equal).

## Data Availability

The data used to support the findings of this study are available from the corresponding author upon request.
